# Liver Cancer and Aflatoxin: New Information from the Kenyan Outbreak

**Published:** 2005-12

**Authors:** Julia R. Barrett

Millions of people are exposed to aflatoxins, toxic compounds produced by *Aspergillus* molds. These molds infest staple crops such as maize, peanuts, rice, and wheat throughout the world. Outbreaks of aflatoxicosis affecting up to several hundred people at a time have occurred sporadically, most recently in eastern Kenya in early 2004. An investigation of the Kenyan outbreak now yields new information on the risk factors associated with acute aflatoxin poisoning **[*EHP* 113:1779–1783]**.

Chronic low-level exposure to aflatoxins, particularly aflatoxin B_1_, is associated with increased risk of developing liver cancer, impaired immune function, and malnutrition. Acute high-level exposure, which is less common, causes early symptoms of diminished appetite, malaise, and low fever. Later symptoms, including vomiting, abdominal pain, and hepatitis, signal potentially fatal liver failure.

The Kenyan outbreak followed a poor harvest of maize that had been damaged and made susceptible to mold by drought. Furthermore, to guard against theft of the meager harvest, people stored the maize in their homes, which were warmer and moister than the granaries where it was usually stored. From January to June 2004, 317 people sought hospital treatment for symptoms of liver failure, and 125 died. Health officials ruled out viral liver diseases; suspecting acute aflatoxin poisoning, they examined maize samples and found aflatoxin B_1_ concentrations as high as 4,400 parts per billion (ppb), 220 times the Kenyan limit for food.

Researchers conducted a case–control study using records for 40 patients (cases) who had been hospitalized with acute jaundice during late May and early June and 80 randomly selected controls. Jaundice is a nonspecific symptom of liver damage.

Participants or family members completed questionnaires targeting maize quality, storage, preparation, and consumption. The researchers collected 1-kilogram samples of maize from households that still had grain left over from the time of the outbreak for measurement of aflatoxin concentrations. Blood samples from 29 patients and 62 controls were analyzed for concentrations of aflatoxin B_1_–lysine albumin adduct, a marker of aflatoxin exposure. The researchers also tested blood from 18 patients and 54 controls for hepatitis B surface antigen, an indicator of hepatitis B infection. In people with chronic low-level aflatoxin exposure, this virus enhances the risk of developing liver cancer.

Maize from patients’ homes contained significantly higher amounts of aflatoxin (with a geometric mean of 354.5 ppb) compared to control households (with a geometric mean of 44.1 ppb). Patients’ serum aflatoxin adduct concentrations, which were comparable to those measured in previous outbreaks, were nearly 10 times higher than those of controls. Further, patients who died had higher blood levels of adducts than those who survived. Forty-four percent of the patients tested positive for hepatitis B, compared to 7% of controls.

These analyses, with their greater level of detail, are the first to quantify the association between concentrations of aflatoxin in food, exposure history, concentrations of serum aflatoxin adducts, and acute aflatoxin poisoning. This study is also the first to quantify the independent association between hepatitis B infection and the effects of acute aflatoxin poisoning. The researchers suggest that monitoring both aflatoxin concentrations in crops and the incidence of acute jaundice could permit earlier recognition of food contamination and help prevent an outbreak from becoming widespread. Further, they suggest that future use of blood tests for aflatoxin B_1_–lysine albumin adducts could serve to diagnose aflatoxin poisoning and to gauge the success of measures for reducing aflatoxin exposure.

## Figures and Tables

**Figure:**
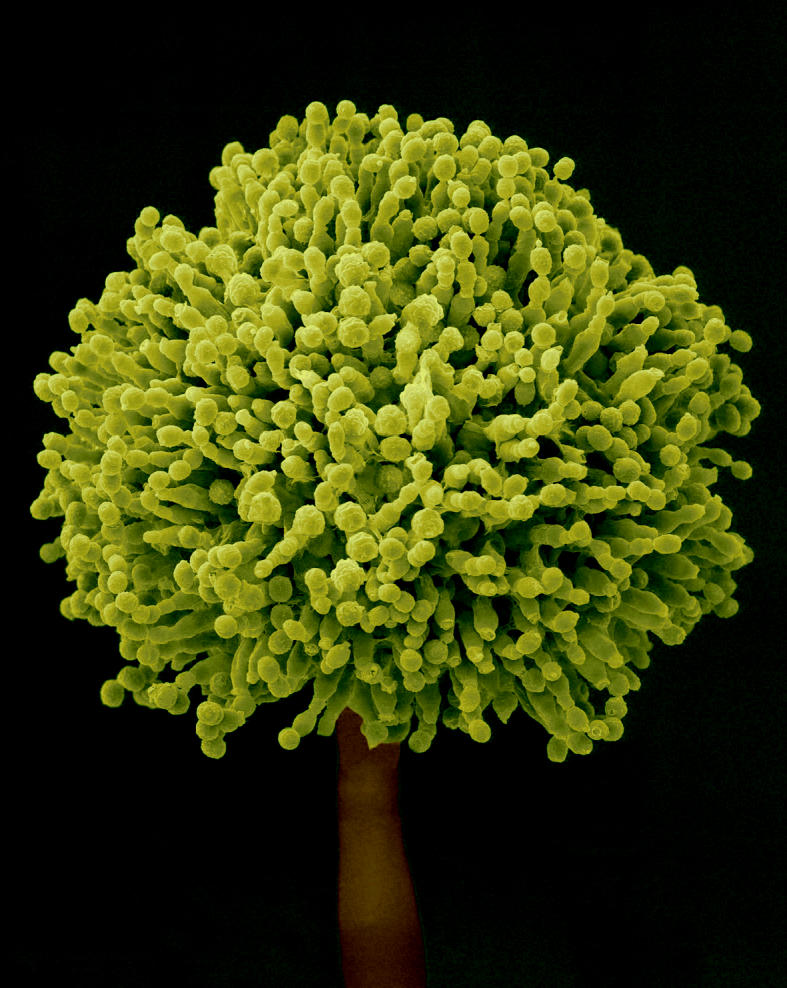
Tiny killer. Chronic low-level exposure to aflatoxins produced by *Aspergillus* molds (such as *A. flavus*, above) is associated with increased risk of liver cancer.

